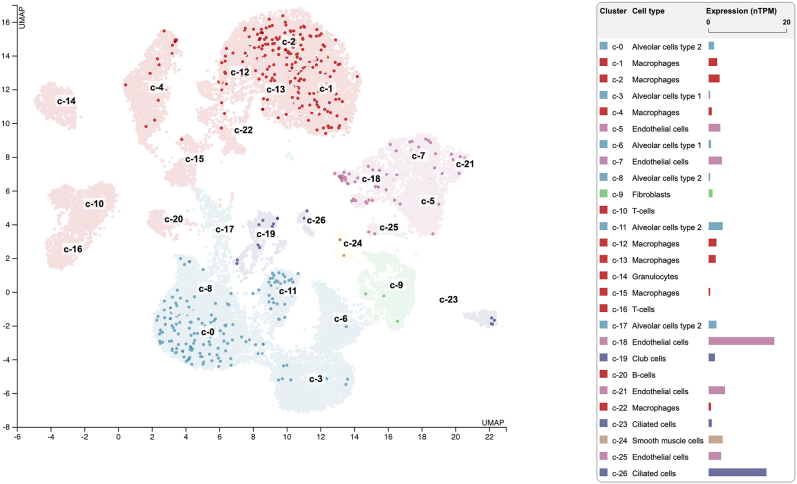# BATF2 inhibits PD-L1 expression and regulates CD8+ T-cell infiltration in non–small cell lung cancer

**DOI:** 10.1016/j.jbc.2023.105302

**Published:** 2023-09-29

**Authors:** Junwei Liu, Jie Li, Zhan Tuo, Weidong Hu, Jun Liu

**Affiliations:** 1 Department of Thoracic Surgery, Zhongnan Hospital of Wuhan University, Wuhan, China; 2 Department of Immunology, University of South Florida, Tampa, Florida, USA; 3 Department of Immunotherapy, Affiliated Cancer Hospital of Zhengzhou University & Henan Cancer Hospital, Zhengzhou, Henan, P.R. China

**Keywords:** BATF2, immunotherapy, lung cancer, PD-L1, ZEB2

## Abstract

Immune checkpoint blockades have made huge breakthrough among some cancer types including lung cancer. However, only a small proportion of patients will benefit from immune checkpoint blockades; other patients have no or minor response to immunotherapy. The underlying mechanisms and efficient biomarkers to predict immunotherapy resistances remain unclear and lacking. In this study, BATF2 knockout mice, human xenograft mice, were used for *in vivo* studies. Relevant RNA and protein levels were analyzed by RT–quantitative PCR and Western blotting. As a result, we found that the expression of BATF2 is negatively correlated with expression of programmed death-ligand 1 in the plasma of patients. Mechanically, we showed that BATF2 inhibits programmed death-ligand 1 expression in cancer cells by inhibiting the PI3K–AKT pathway where ZEB2 plays an important role in this process. Based on bioinformatics analysis, we found that the function of BATF2 in promoting antitumor immune response in patients with non–small cell lung cancer, which is mediated by BATF2, enhances CD8+ T-cell infiltration as well as activation. The expression of BATF2 from circulating tumor cells and tissues can be serve as an efficient biomarker to predict diagnosis, prognosis, and immunotherapy efficacy.

Lung cancer is still the leading cancer-related death in males and second leading cause of death in females (1, 2). While lung cancer has different subtypes, non–small cell lung cancer (NSCLC) still accounts for nearly 85% in whole lung cancer cases (3). Except the standard treatments with chemotherapy or irradiation, in the past decade, immune checkpoint blockades (ICBs) targeting programmed death protein 1, programmed death-ligand 1 (PD-L1), or cytotoxic T lymphocyte antigen 4 have revolutionized the treatment of NSCLC and rapidly become the first-line treatment for patients with advanced NSCLC (4, 5, 6). However, only 10 to 20% of patients will benefit from ICB, and the majority of those patients is still resistant or have minor response to immune therapies, and underlying mechanisms remain unclear. Therefore, new mechanism studies and predictive biomarkers are urgently needed to better stratify the patient population to assist better clinical practice (7, 8).

BATF2, one of the transcription factor from basic leucine zipper transcription factor family, has been shown to be related to multiple types of cancers (9, 10, 11, 12, 13, 14). BATF2 has been reported as an independent prognostic factor in gastric cancer patients, and high BATF2 expression significantly suppressed gastric cancer growth and metastasis (13). Similarly, in colon cancer, BATF2 was found to be downregulated, and high BATF2 was related with epithelial-to mesenchymal (EMT) transition (9, 12). Importantly, from our previous work, we found that BATF2 deficiency facilitates EMT transition, which contributes to lung cancer metastasis by regulating β-catenin–glycogen synthase kinase 3β pathway (15).

BATF2 can express not only on cancer cells but also in immune cell population including T cells, B cells, and other myeloid populations. As a transcriptional factor, BATF2 has been involved in immune cell differentiation and function. Considering the important role of BATF2 in immune cells, here we investigated whether BATF2 can affect antitumor immune response. Our findings showed that BATF2 inhibits PD-L1 in cancer cells through PI3K–AKT pathway. In addition, increasing BATF2 expression promotes T-cell infiltration as well as its activation in patients. These results demonstrated that BATF2 is associated with tumor immunity in NSCLC and may perform as a prognostic factor to predict patients’ response to immunotherapies.

## Results

### BATF2 inhibits the expression of PD-L1 in NSCLC cell lines

**Figure 1:**
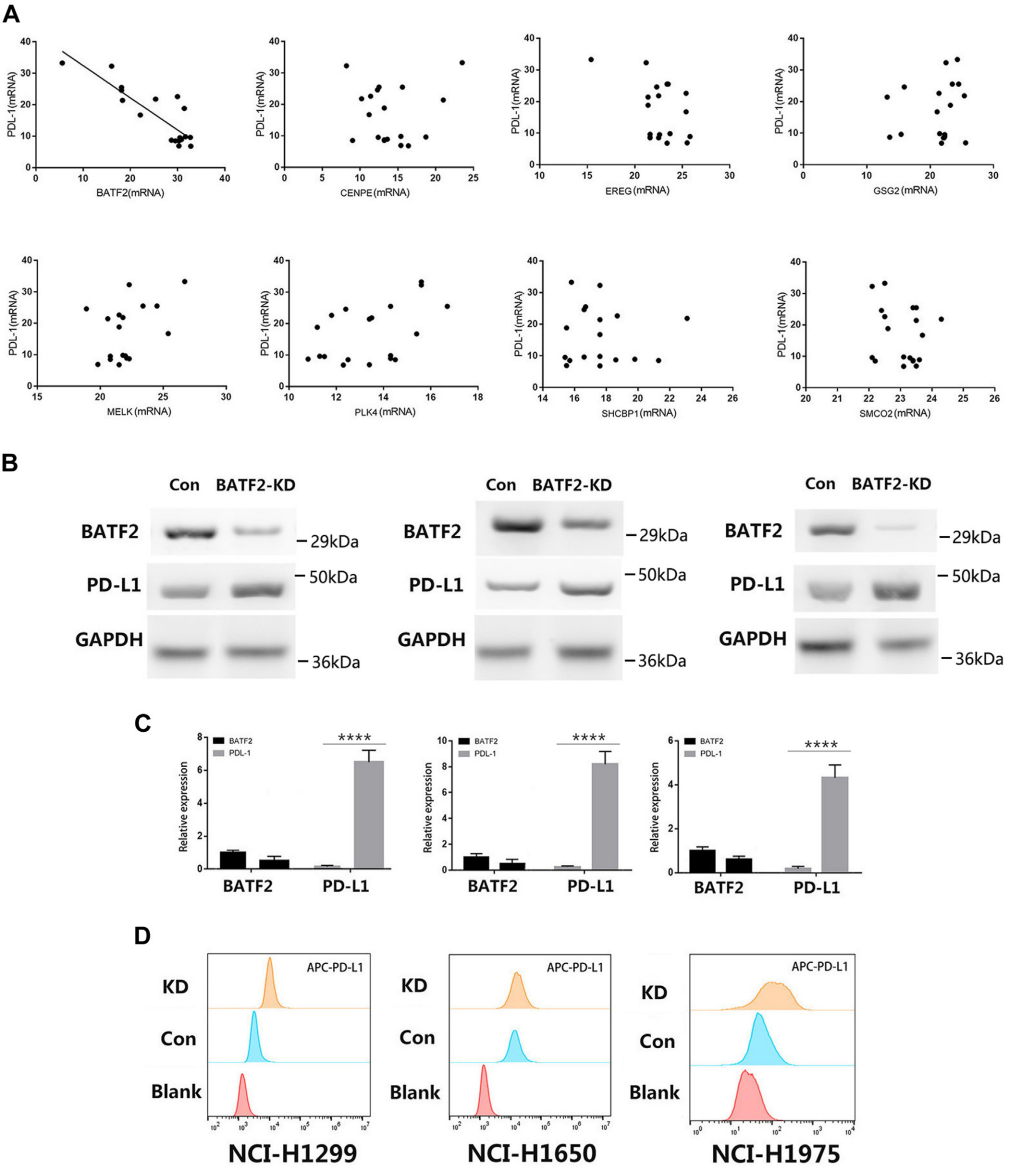
**Expression of BATF2 in plasma is negatively correlated with the expression of PD-L1.***A*, the mRNA expression of several genes in plasma was detected by RT–qPCR, and the correlation between the mRNA of these genes and PD-L1 in lung cancer was assessed using the Spearman rank correlation. *B*, the expression of BATF2 and PD-L1 by Western blotting after transfecting of NCI-H1299, NCI-H1650, and NCI-H1975 cells with either empty vector or BATF2 siRNA. *C*, the mRNA expression of PD-L1 in NCI-H1299, NCI-H1650, and NCI-H1975 cells after knocking down *BATF2*. ∗∗∗∗*p* < 0.0001 *via* unpaired Student’s *t* test. *D*, the expression of PD-L1 on the cell membrane was detected by flow cytometry. PD-L1, programmed death-ligand 1; qPCR, quantitative PCR.

To explore the relationships between BATF2 and PD-L1 expression, we initiated an observational, analytical, open retrospective study (ChiCTR2000033546) involving 18 stage IV lung cancer patients who were about to receive immunotherapy. The majority of the patients were males, and all the patients had stage IV lung cancer (Table S1). Thus, it can be inferred that BATF2 expression in plasma is related to PD-L1 expression in NSCLC patients (Fig. 1*A*). Next, we investigated the role of BATF2 *in vitro* by using human lung cancer cell lines (NCI-H1299, NCI-H1650, and NCI-H1975). As expected, knocking down BATF2 upregulated PD-L1 expression both on RNA and protein levels (Fig. 1, *B* and *C*). Interestingly, we also observed that BATF2 knockdown (KD) upregulated PD-L1 expression on the cell membrane (Fig. 1*D*). In sum, BATF2 is negatively correlated with the expression of PD-L1 in patients with NSCLC, and BATF2 inhibits PD-L1 expression both in mRNA and protein levels.

### BATF2 inhibits PD-L1 expression through downregulating ZEB2

**Figure 2:**
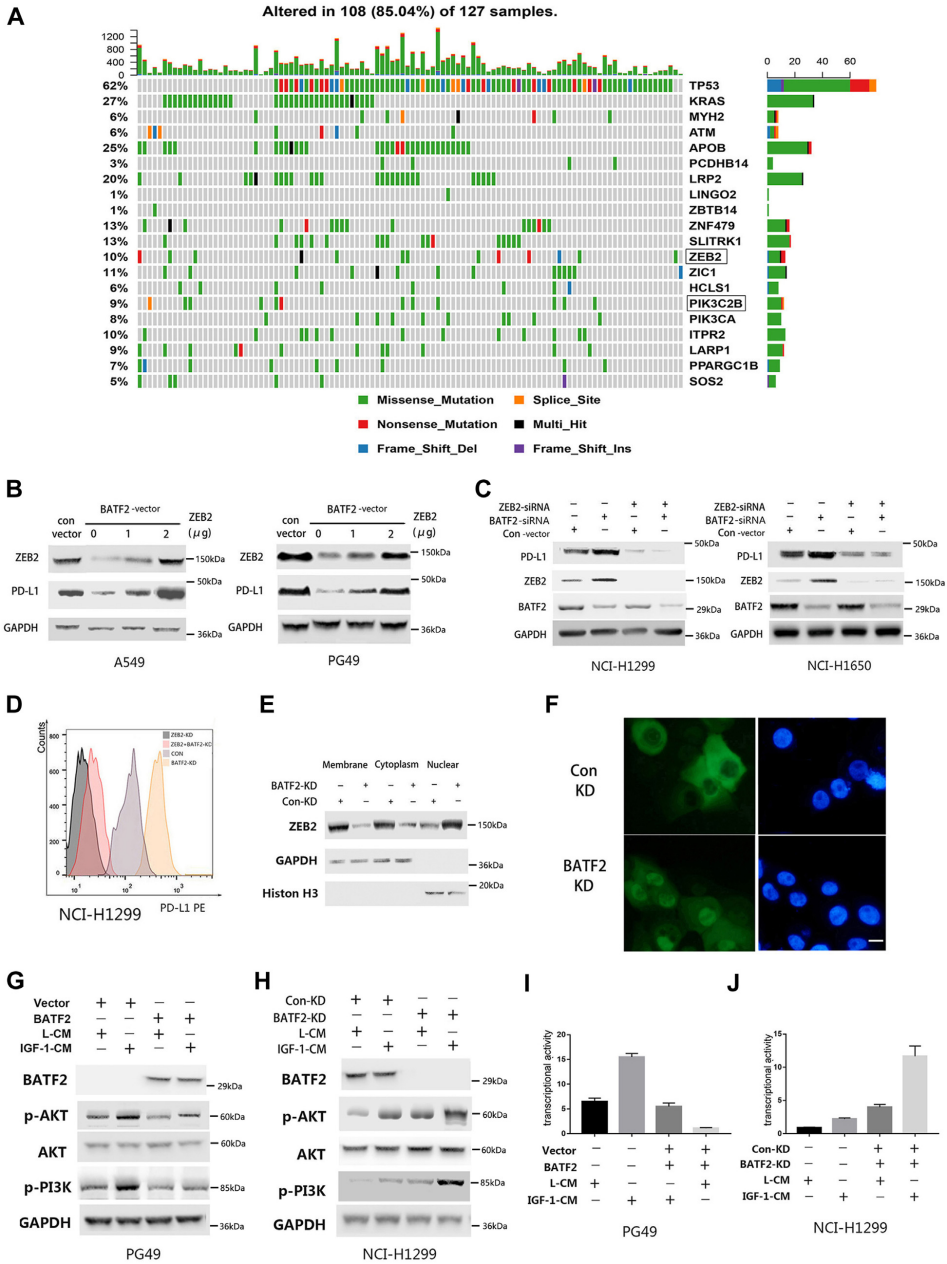
**BATF2 inhibits PD-L1 expression through downregulating ZEB2.***A*, somatic cell mutation rates in LADC with BATF2 upregulation. *B*, the expression of ZEB2 and PD-L1 after cotransfecting increasing levels of ZEB2 cDNA and BATF2-vector into A549 and PG49 cells. *C*, expression of PD-L1, ZEB2, and BATF2 after single knocking down or double knocking down by ZEB2 siRNAs and BATF2 siRNAs in NCI-H1299 and NCI-H1650. *D*, expression of PD-L1 in NCI-H1299 by flow cytometry. *E*, subcellular localizations of ZEB2 after knocking down BATF2 were by Western blotting. *F*, representative image of ZEB2 location by IF. Scale bar represents 20 μm. *G*, after exogenous addition of BATF2, the expression of BATF2, p-AKT, AKT, and p-PI3K in PG49 cells cultured in CM or IGF-1 CM. *H*, after knocking down *BATF2*, the expression of BATF2, p-AKT, AKT, and p-PI3K in NCI-1299 cells cultured in CM or IGF-1 CM. *I* and *J*, luciferase activity in PG49 and NCI-1299 cells after same treatment as mentioned above. cDNA, complementary DNA; CM, conditioned media; IF, immunofluorescence; IGF-1, insulin-like growth factor-1; LADC, lung adenocarcinoma; PD-L1, programmed death-ligand 1.

Cancer cells tend to be unstable and have somatic mutations and copy number alterations; therefore, we wondered whether BATF2-regulated PD-L1 expression was mediated by signaling pathway alteration during the unstable process. After comparing the mutation frequencies of samples with high BATF2 expression, the highest mutation rates, except for TP53, were observed in ZEB2 in the samples with high BATF2 expression (Fig. 2*A*). ZEB2 is a DNA-binding transcription factor; previous studies have shown that ZEB2 can induce tumor cells to express PD-L1 and then escape immune surveillance (16, 17). Thus, we hypothesized that BATF2 may inhibit PD-L1 expression by downregulating ZEB2. To verify this hypothesis, we explored whether the inhibiting effect of BATF2 could be reversed by ZEB2 overexpression. Interestingly, the increased levels of ZEB2 complementary DNA could upregulate the protein expression of PD-L1 (Fig. 2*B*). However, BATF2 KD–induced upregulation of PD-L1 protein could be abrogated by knocking down ZEB2 (Fig. 2, *C* and *D*) indicating BATF2 is dependent on ZEB2 to regulate PD-L1 expression. ZEB2 is regulated by upstream pathways, including PI3K–AKT (18, 19), mitogen-activated protein kinase, and transforming growth factor-β (20, 21). Interestingly, the reduced levels of cytoplasmic ZEB2, membrane-associated ZEB2, and accumulation of nuclear translocation of ZEB2 were found in BATF2-KD cells suggesting the transcriptional activation of ZEB2 (Fig. 2, *E* and *F*). Utilizing the somatic mutations pinpointed within the cBioPortal datasets, it was observed that there was a greater prevalence of mutations in ZEB2 and PIK3C2B within specimens exhibiting elevated BATF2 expression. As a result, we delved into an inquiry regarding the potential involvement of the PI3K–AKT signaling pathway in this phenomenon. We used insulin-like growth factor-1 (IGF-1), which signals *via* PI3K cascades. Therefore, treating cells with CM with IGF-1 protein can induce the phosphorylation of AKT and PI3K (22, 23). We found CM with IGF-1 only slightly elicited AKT–PI3K activation when exogenous BATF2 was added into PG49 cells (Fig. 2*G*), whereas activity was significantly increased when knocking down BATF2 into NCI-H1299 cells (Fig. 2*H*) suggesting that BATF2 can inhibit PI3K–AKT activity and therefore inhibit ZEB2 expression. Similar results were also shown based on LRP assay that BATF2 could significantly decrease the transcription level of ZEB2 induced by IGF-1 CM (Fig. 2, *I* and *J*), suggesting that BATF2 regulated ZEB2 through AKT–PI3K signaling pathway. In conclusion, BATF2 that inhibits the expression of PD-L1 is ZEB2 dependent.

### BATF2 increases PD-L1 in orthotopic tumor model

**Figure 3:**
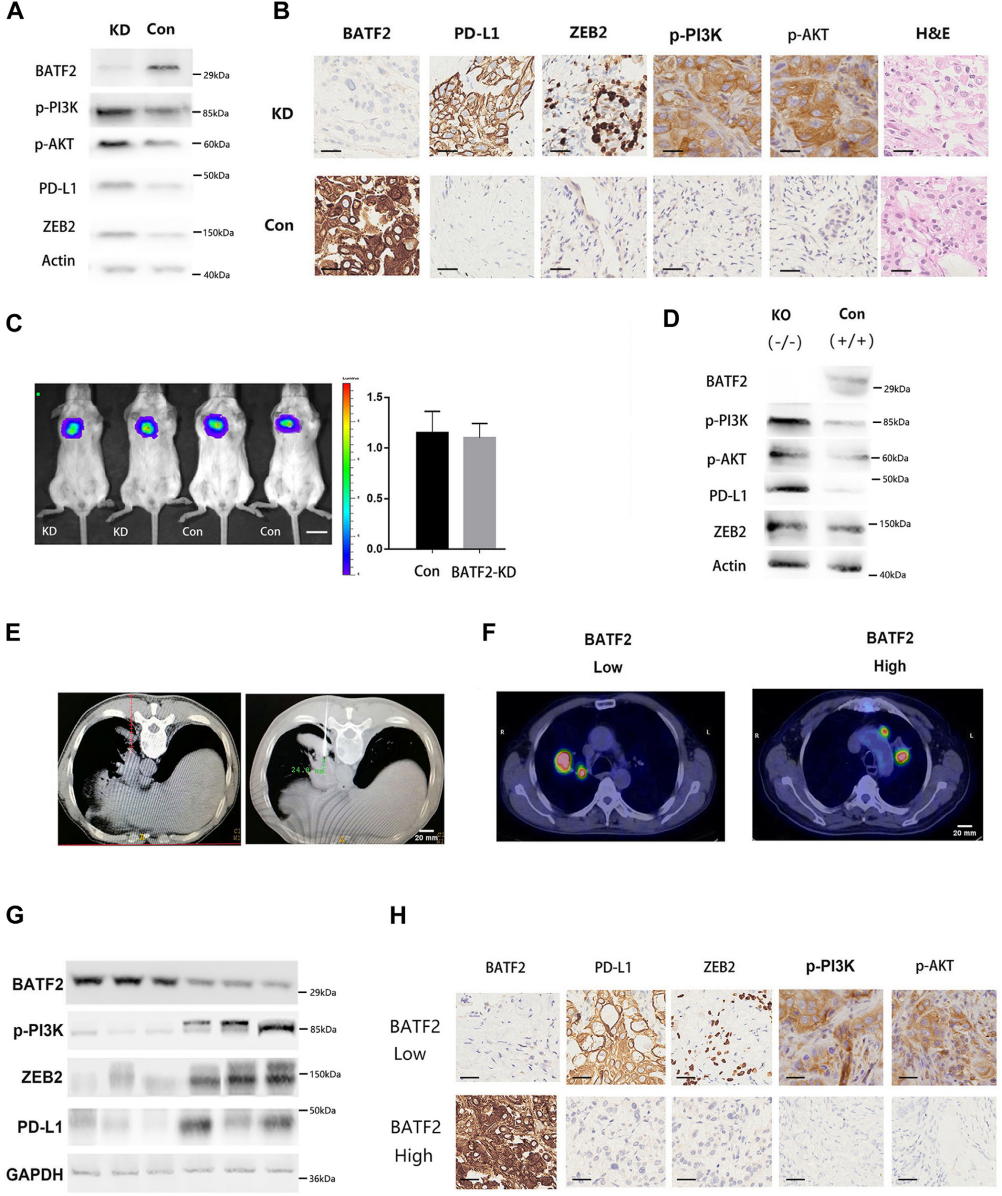
**BATF2 modulates PD-L1 *in vivo.****A*, the protein levels of PD-L1, p-PI3K, p-AKT, and ZEB2 in BATF2 KD tumor by Western blot. *B*, representative immunohistochemistry images of PD-L1, p-PI3K, p-AKT, ZEB2, and BATF2 from orthotopic NCI-H1650-BATF2 KD tumor. Scale bar represents 100 μm. *C*, tumor size was measured by BLI in nude mice with NCI-H1650-KD tumor. Scale bar represents 1 cm. *D*, expression of PD-L1, p-PI3K, p-AKT, PD-L1, and ZEB2 in normal lung tissue from BATF2 KO mice. *E*, representative images of sample collection under CT-guided percutaneous needle biopsy. Scale bar represents 2 cm. *F*, tumor volume was determined by positron emission tomography/CT. Scale bar represents 2 cm. *G*, the protein levels of BATF2, PD-L1, p-PI3K, and ZEB2 in BATF2 low (n = 3) and high (n = 3) patients’ lung cancer tissues by Western blotting. *H*, immunohistochemistry staining of PD-L1, ZEB2, p-PI3K, and p-AKT in patient samples with low or high BATF2 expression. Scale bar represents 100 μm. BLI, bioluminescence imaging; PD-L1, programmed death-ligand 1; KD, knockdown.

To determine whether BATF2 could modulate PD-L1 *in vivo*, we first tested the expression of PD-L1 in nude mice with orthotopic lung cancer. Similar to *in vitro* data, we found that BATF2-KD tumor-bearing mice had upregulated expression levels of PD-L1, ZEB2, and p-AKT–PI3K (Fig. 3, *A* and *B*). To our surprise, knocking down BATF2 did not reduce tumor burden in our model indicating that the tumor control efficacy we observed in patients may be due to host since nude mice is immune compromised (Fig. 3*C*). Next, we used BATF2-knockout (BATF2^−/−^) mice to check under physiological conditions, whether PD-L1 expression was altered in lung epithelial cells. We found that PD-L1 and AKT–PI3K–ZEB2 pathway expression was upregulated (Fig. 3*D*). To further verify the association between BATF2 and PD-L1 expression, an observational, analytical, open retrospective study was carried out. Stage IV patients were selected according to the TNM staging system. Six lung cancer samples were collected, and the size of tissue ranged from 3 to 10 cm, which were analyzed by positron emission tomography/computed tomography (Fig. 3, *E* and *F*). Importantly, when patients lost BATF2 expression, the expression of p-PI3K, ZEB2, and PD-L1 increased as well (Fig. 3, *G* and *H*), which is consistent with our *in vitro* and mouse data.

### BATF2 predicts better antitumor immunity in NSCLC

**Figure 4:**
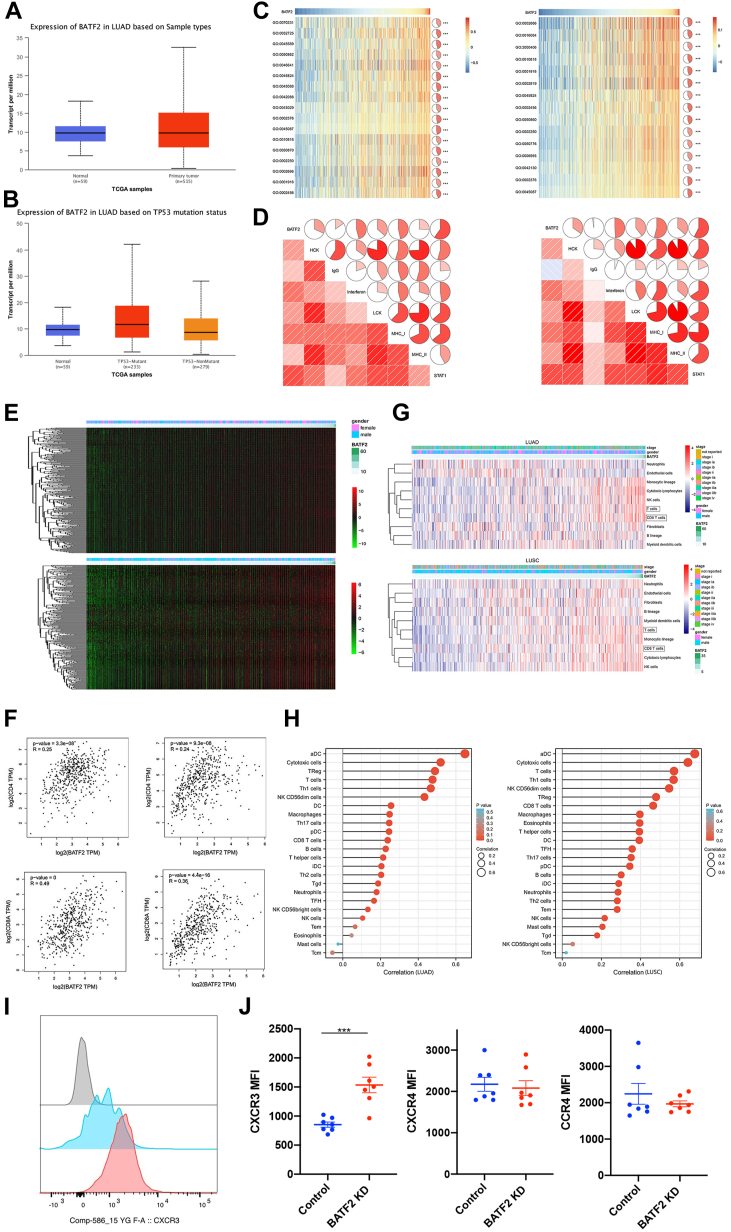
**Bioinformatics analysis of BATF2 in NSCLC**. *A*, expression of BATF2 is higher in LADC compared with normal tissue. *B*, expression of BATF2 is higher in patients with p53 mutation. *C*, GO enrichment of the positively correlated bioprocess in LADC and LSSC. *D*, BATF2 expression was positively correlated with most immune-associated genes in LADC and LSSC by heatmap. *E*, the relationship between BATF2 and inflammatory activity in LADC and LSSC. *F*, BATF2 was positively correlated with CD4+ T cells and CD8+ T cells in LADC and LSSC. *G* and *H*, BATF2 is positively correlated with antitumor immune cell infiltration in LADC and LSSC. *I*, representative histograms showing CXCR3 expression by tumor CD8+ T cells in control and BATF2 KD tumor. *J*, mean fluorescence intensity (MFI) of surface CXCR3 on tumor T cells. Data pooled from two experiments. CXCR3, C–X–C chemokine receptor 3; GO, Gene Ontology; KD, knockdown; LADC, lung adenocarcinoma; LSSC, lung squamous cell carcinoma; NSCLC, non–small cell lung cancer.

As reported previously, BATF2 plays a crucial role in regulating immune cell biological functions (24, 25). To our surprise, we found that the expression of BATF2 is higher in primary tumor compared with normal tissue; whether there is loss of function of BATF2 in those patients’ tumor needs further investigation (Fig. 4*A*). In addition, tumor with p53 mutation tends to have higher expression of BATF2. To investigate whether BATF2 also affects immune cells, we selected genes from LADC and LSSC subtypes that are strongly correlated with BATF2 by Pearson correlation analysis (Pearson's *R* > 0.3) using the TCGA datasets. Interestingly, the results demonstrated that the genes that positively correlated with BATF2 were also responsible for immune and inflammatory responses such as major histocompatibility complex I (MHCI), MHCII, and STAT1 (Fig. 4, *C* and *D*). Therefore, BATF2 expression in NSCLC was mainly associated with immune responses. To further analyze the functions of BATF2 in antitumor immune responses, seven immune-related metagenes were detected. The findings demonstrated that BATF2 expression was positively correlated with all seven metagenes (Fig. 4*E*). In addition, BATF2 overexpression was associated with the activation of signal transduction in antigen-presenting cells, B lymphocyte, macrophages, and T cell-related metagenes. Taken together, our findings indicate that BATF2 also plays critical roles in modulating the immune and inflammatory functions of LADC and LSSC subtypes. Dysregulation of BATF2 is observed in different tumors, which can contribute to tumor progression. T cells, especially CD8+ T cells, play an important role for antitumor immunity (26); therefore, we investigate the role of BATF2 and T cells. As expected, BATF2 was also strongly related to different subtypes of T cells (Fig. 4*F*). To be noticed, high expression of BATF2 also promotes infiltration of antitumor immune cells, mainly dentritic cells, cytotoxic T cells, and NK cells in both lung cancer subtypes (Fig. 4, *G* and *H*). To further characterize the underlying mechanism for T-cell infiltration, we tested several cytokine receptors that are well known related to CD8 T-cell infiltration including C–X–C chemokine receptors 3 and 4 and C–C chemokine receptor type 4 (CCR4); only surface expression of C–X–C chemokine receptor 3 significantly increased in tumor-infiltrating CD8+ T cells in BATF2 KD tumor (Fig. 4, *I* and *J*). As a conclusion, BATF2 expression in tumor is positively correlated with antitumor immune cell infiltration.

## Discussion

Immunotherapy emerges as an important role in the treatment regimens. Pembrolizumab and atezolizumab have been used as the first line for lung cancer patients. However， not all the patients even meet with the criteria that high PDL-1 expression (≥50%) but no epidermal growth factor receptor or anaplastic lymphoma kinase mutation will benefit from ICBs. Hence, there is an urgent requirement to gain a deeper comprehension of the mechanism involved and identify suitable biomarkers for predicting both acquired and inherent immunotherapy resistance.

In our research, we found that BATF2 expression is negatively correlated with the expression of PD-L1. Knocking out BATF2 *in vitro* significantly increased the expression of PD-L1. BATF2 has been previously reported as a novel tumor suppressor gene that inhibits the growth of cancer cells through repression of hepatocyte growth factor receptor/MET signaling. To further understand how BATF2 regulates PD-L1, using the TCGA database, we found that lung cancer patients with high BATF2 are negatively correlated with ZEB2 and PI3K signaling pathway. Interestingly, as an important EMT-related transcription factor, ZEB2 has been reported to regulate PD-L1 expression as well as been regulated by PI3K–AKT signaling pathway. Therefore, we hypothesize that BATF2 regulates the expression of PD-L1 through ZEB2 and PI3K–AKT signaling pathway. We confirmed this hypothesis *in vitro* by using human lung cancer cell lines and found that increasing the expression of BATF2 in lung cancer cells can prevent ZEB2 from entering the nucleus. In addition, in patients’ samples, we also found increased p-AKT, p-PI3K, ZEB2, and PD-L1 levels from lung cancer patients with low BATF2 expression. Interestingly, nude mice with BATF2-KD tumors expressed higher levels of PD-L1, ZEB2, and p-AKT–PI3K; however, there are no differences of tumor growth in this immune-compromised model where in patient samples, BATF2 is correlated with better prognosis, indicating that BATF2 on host immune cells plays an important role for antitumor immunity. To investigate whether BATF2 affects the immune response, using TCGA database, we found that the increase in BATF2 expression was positive related to antitumor immune cell infiltration especially with dentritic cell, cytotoxic CD8 T cells, and NK cells, which may be mediated by increasing inflammatory cytokine production such as interferon-gamma as well as antigen presentation (MHCI and MHCII). Further studies will be required to study how BATF2 can affect immune infiltration. Is it possible through metabolism reprograming or affecting cell proliferation or tumor immune landscape especially macrophages that has been reported and has decent amount of BATF2 expression in basal level (Fig. S1)?

**Figure 5:**
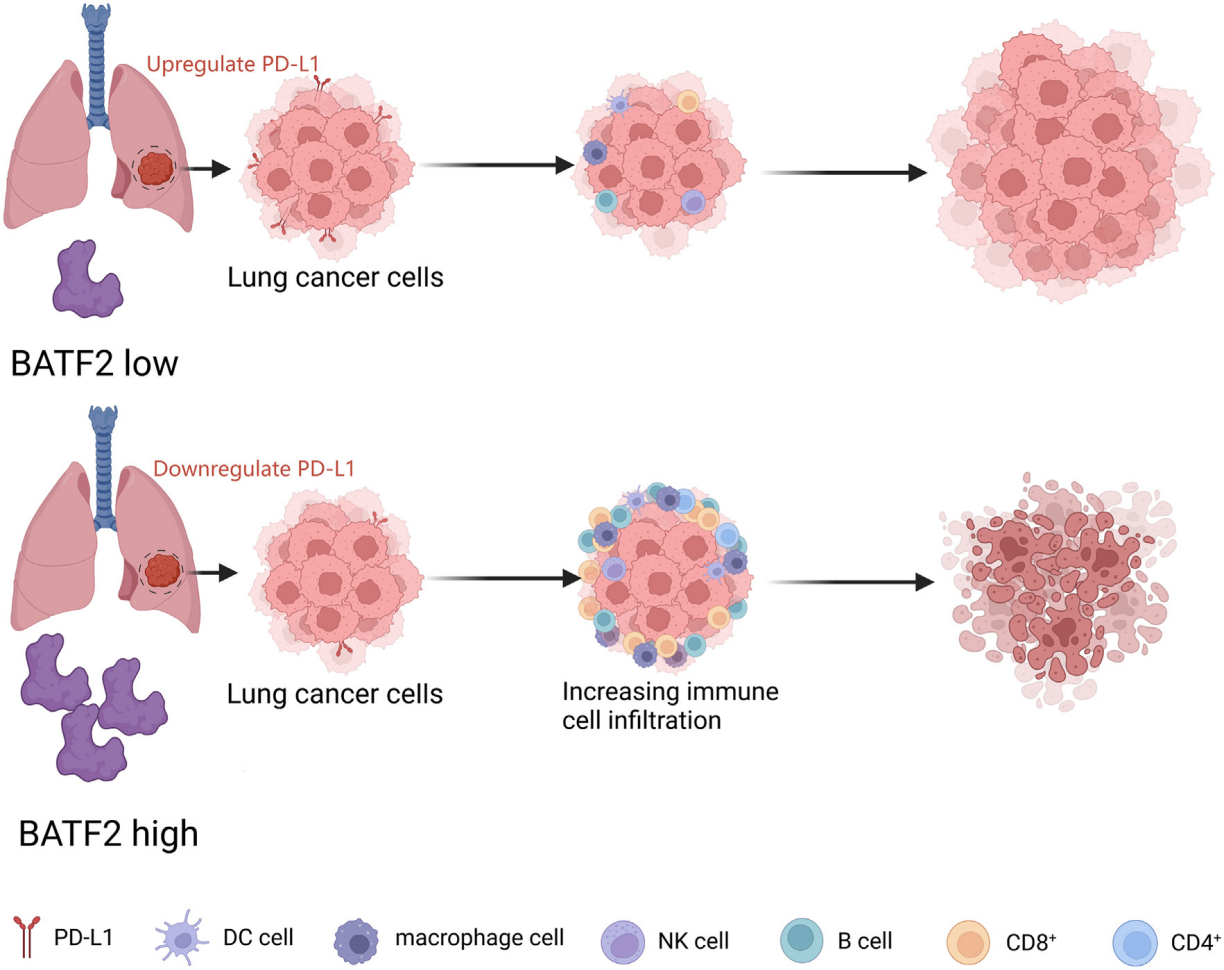
**BATF2 inhibits PD-L1 expression and promotes tumor immune infiltration in lung cancer**. Patients with high BATF2 expressing tumor will downregulate PD-L1 expression in lung cancer cells, which further promote more immune cell infiltration especially CD8+ T cells to kill the tumor. However, with low BATF2 expression, the tumor is immune cold, and even when high PD-L1 is expressed, the immunotherapy may fail because of few immune cell infiltrations. PD-L1, programmed death-ligand 1.

In summary, we found that BATF2 is a novel regulator of PD-L1 and that BATF2 inhibits PD-L1 expression in lung cancer as well as promotes tumor immune infiltration. Therefore, BATF2 can be a potential biomarker to evaluate the efficacy of ICBs, low BATF2 expression indicating higher expression of PD-L1, and tracking dynamic BATF2 expression level from plasma may serve as an indicator for PD-L1 expression in tumor instead of multiple repeats of sample biopsies from patients (Fig. 5).

## Experimental procedures

### Data collection and patient selection

The datasets were obtained from three sources. First, several datasets, such as the transcriptomic expression data of lung squamous cell carcinoma (LSSC, n = 484) and lung adenocarcinoma (LADC, n = 566), mutation data respective to RNA-sequencing data, and reverse-phase protein array–based proteomic data, were retrieved from The Cancer Genome Atlas (TCGA). The level-3 TCGA data were employed for subsequent analyses. The cBioPortal for Cancer Genomics was used to obtain the second data source, where a portion of the copy number–altered genome data was downloaded. The third data source was from the Wuhan Union Hospital, where clinical lung cancer (LC) tissues were collected. Tumor and blood specimens were collected from LC patients prior to the treatment *via* computed tomography–guided percutaneous needle biopsy. The circulating tumor cell (CTC) biopsy system was used to examine CTCs from plasma, and BATF2 was detected by RT–quantitative PCR (qPCR). The study protocol was approved from the Ethical Committee of the Huazhong University of Science and Technology. All patients provided a written informed consent.

### Bioinformatics analysis

The GraphPad Prism 7 (GraphPad Software, Inc), SPSS 23 (IBM SPSS, Inc), and R 3.4.3 (R Foundation for Statistical Computing) software were used to conduct statistical test and draw images. Patient’s data from both the Chinese Glioma Genome Atlas and TCGA datasets were analyzed using Q4. Correlations between BATF2 expression and expression of other genes and immune cells were evaluated by Pearson’s correlation coefficient (*r*). The correlations for BATF2 were regarded as significant when the absolute *r* value was greater than 0.3. Gene annotation and pathways were examined with DAVID 6.8 (DAVID Bioinformatics Resources, ncifcrf.gov) Gene Ontology analysis was conducted to assess the biologic function of BATF2. Gene set variation analysis was performed to explore the relationships between BATF2 and candidate gene function as well as immune cell types to further verify the effect of BATF2 expression on a specific subset of immune cells. The thresholds employed for selecting immune cells correlated with BATF2 were >0.45 (absolute *r* value) and <0.05 (*p* value).

### Cell culture and sample collection

A549, NCI-H1299, NCI-H1650, and NCI-1975 cells were cultured in 10% fetal bovine serum (Gibco)–containing RPMI1640 Lewis medium (Gibco). PG49 cells were cultured in 10% fetal bovine serum–containing Dulbecco's modified Eagle's medium (Gibco). All cells were incubated at 37 °C and 5% CO_2_.

### Antibodies, plasmids, and reagents

Plasmids for ZEB2 and BATF2 were supplied by Sigma. For transfection experiments, the cells (5 × 10^5^ cells/well) were grown in 6-well plates (Costar) until reaching 70 to 80% confluency. Lipofectamine PLUS (Invitrogen) was used for cell transfection by following the kit’s protocols. L-cell-conditioned medium (CM) and insulin-like growth factor 1-CM were harvested in accordance with the instructions provided by the American Type Culture Collection and then incubated for 24 h in the cells. The primary antibodies used were as follows: anti-BATF2 (CST), anti-PD-L1 (CST), anti-ZEB2 (Sigma), anti-PI3K (CST), anti-AKT (Sigma), anti–phospho-AKT (CST), anti–phospho-PI3K (p85, Y458) (CSK), and anti-GAPDH (Abcam). The siRNA-BATF2 for use in mice and humans was obtained from Abcam.

### Western blot analyses

Total protein was extracted using the radioimmunoprecipitation assay buffer containing phosphatase and protease inhibitors. Membrane, nuclear, and cytosolic protein extractions were conducted by following the kit’s protocols. The protein concentrations were measured using the Bradford method with 0.1 mg/ml bovine serum albumin (BSA) as the standard. After separation through 10% SDS-PAGE, the protein specimens were transferred onto a polyvinylidene difluoride membrane. The membrane was blocked for 2 h with 5% nonfat milk in Tris-buffered saline with Tween-20 (10 mmol/l Tris–HCl [pH 7.4], 150 mmol/l NaCl, and 0.1% Tween-20). After incubation overnight at 4 °C with primary antibodies, the membrane was incubated again with secondary antibodies for 1 h. Finally, enhanced chemiluminescence reagent was used to visualize the protein blots.

### Flow cytometric analysis of cell lines

Cells (1 × 10^6^ cells) were collected and blocked by FC blocking buffer (BioLegend), and then the cells were stained with allophycocyanin-conjugated PD-L1 (BD Biosciences) for 30 min on ice; after washing three times with PBS, cells were analyzed immediately with flow cytometry canto with FACSDiva Software (BD Biosciences).

### Confocal fluorescence microscopy

BATF2 siRNA or control was used to transfect the cells for 48 h. After fixing in formaldehyde (4%), the cells were permeabilized with 0.1% Triton X-100 (Sigma) in PBS for 5 min and blocked with PBS supplemented with 0.5% BSA for 1 h. Then the cells were incubated with primary antibodies diluted in the blocking buffer overnight at 4 ˚C. Washing three times in PBS, the cells were stained with FITC-immunoglobulin G (Molecular Probes). The nuclei were stained with 4′,6-diamidino-2-phenylindole (5 min). Olympus confocal microscope was used to perform confocal immunofluorescence microscopy (magnification 60×).

### Luciferase reporter phage gene assay

The cells were grown in 24-well plates and transfected with ZEB2, a firefly luciferase reporter phage (LRP) construct (200 ng FOP or TOP) and an internal control pRL-SV40 (1 ng). After transfection for 2 days, the cell extract was prepared. The Dual-Luciferase Reporter Assay System (Promega) was used to evaluate the LRP activity.

### RT–qPCR assays

TRIzol reagent (Invitrogen) was utilized to extract total RNA. Complementary DNA synthesis was performed using the SYBR Premix Ex TaqTM II (Takara Bio). RT–qPCR assays were carried out on a StepOnePlus Real-Time PCR system (Applied Biosystems) using the SYBR Green Mastermix (Takara Bio). The primers were obtained from Wuhan BOSTER company. To assess the relative mRNA expression, triplicate measurements of each sample were normalized to GAPDH.

### Histology and immunohistochemistry

After removing tumors, weighing, and fixing in formalin (5%), the tissues were paraffin embedded. Sections were deparaffinized in xylene and dehydrated in graded alcohol. Antigen retrieval was performed by boiling slides for 20 min in sodium citrate buffer (pH 6.0), and slides were blocked using blocking buffer (PBS supplemented with 0.5% BSA) for 1 h. Then incubating with primary antibodies overnight at 4 °C. After incubation with antimouse immunoglobulin G secondary antibody (Invitrogen), the tissues were stained using the ABC staining kit (Santa Cruz Biotechnology). The LC tissues were rinsed with PBS, inflated, and fixed in formalin (10%). After paraffin embedding and sectioning at 5 μm thickness, H&E staining was routinely conducted.

### Orthotopic mouse model

Ethical approval for animal experiments was obtained from the Institutional Animal Care and Use Committee of China. The lungs of nude mice (male, 6–8 weeks old) were exposed and then injected with 1 × 10^6^ cell suspension of Matrigel (1:1 ratio, 50 μl in total). After injection for 7 days, the surgical staple was removed. The tumor growth and local metastasis were recorded by bioluminescence imaging (BLI).

### BLI assessment

The IVIS Imaging System (Xenogen) was used for BLI assessments. Bioluminescence signals were obtained and analyzed with Living Image and Xenogen software (IVIS Imaging System). In each BLI session, three mice were anesthetized and intraperitoneally injected with 150 mg/kg d-luciferin. After 3 min of injection, the mice were imaged for 10 min.

### CTC

For collecting CTC, blood was consistently taken from cancer patients either prior to or at least 7 days following intravenous therapy. The blood samples were collected in the CellSave Preservative Tube, provided by Veridex LLC. The blood draw tube contained EDTA as an anticoagulant and a cellular preservative. The samples were processed within 72 h of collection using the CellSearch system developed by Veridex LLC. The CellSearch system comprised the CellPrep system and the CellSearch Epithelial Cell Kit. In summary, 7.5 ml of blood were mixed with 6 ml of buffer, centrifuged at 800*g* for 10 min, and then placed on the CellPrep system. The instrument removed the plasma and buffer layer, and ferrofluids were subsequently added. After an incubation period and magnetic separation, any unbound cells and remaining plasma were aspirated. Then relative mRNA level of CTC was tested by RT–qPCR by using Invitrogen TaqMan Gene Expression Cells-to-CT Kit, and the GAPDH has been used as a loading control.

### Statistical analysis

The cutoff point for BATF2 expression was evaluated with X-tile (Yale School of Medicine) software. For growth curves *in vivo*, two-way ANOVA was used to analyze the significance. To compare two groups with equal variance or unequal variance, two-way unpaired *t* test or two-way unpaired *t* test with Welch’s correction was used respectively. For multiple group comparisons, one-way ANOVA was used. All values are shown as mean ± SD. GraphPad was used to perform statistical tests. *p* < 0.05 was deemed statistically significant.

## Data availability

All the data are within the manuscript and Supporting information. All the data are to be shared upon request.

## Supporting information

This article contains supporting information.

## Conflict of interest

The authors declare that they have no conflicts of interest with the contents of this article.

## Figures and Tables

**Supplementary figures:**