# Supplementary material for: BATF2 inhibits PD-L1 expression and regulates CD8+ T-cell infiltration in non–small cell lung cancer
Source: J Biol Chem. 2023 Sep 29;299(11):105302. doi: 10.1016/j.jbc.2023.105302 (PMC10641166; doi:10.1016/j.jbc.2023.105302)
Supplement: Supporting information [file mmc1.docx]

Supplementary Table 1 Patient characteristics

| Characteristic Total  N=18 | |
| --- | --- |
| Age, yr, median(range) | 62.3(27-86) |
| Sex |  |
| Male | 13 |
| Female | 5 |
| Stage |  |
| IVa  IVb | 6  12 |
| Tumor location  Left Lung  Right Lung | 14  4 |
| Histology subtype |  |
| Adenocarcinoma  Squamous cell carcinoma  Small cell lung cancer | 14  3  1 |
